# Consequences of Periprosthetic Fracture After Hemiarthroplasty of the Hip

**DOI:** 10.7759/cureus.109920

**Published:** 2026-05-30

**Authors:** Stian E Svenøy, Tom T Lian, Wender Figved, Frede Frihagen

**Affiliations:** 1 Orthopaedic Department, Oslo University Hospital, Oslo, NOR; 2 Orthopaedic Department, Baerum Hospital, Baerum, NOR; 3 Orthopaedic Department, Østfold Hospital, Kalnes, NOR

**Keywords:** femoral fracture fixation, hemiarthroplasty of the hip, ortho-geriatric, periprosthetic femur fracture, revision hip arthroplasty, surgical and non-surgical treatment

## Abstract

Introduction

Low-energy femoral neck fracture (FNF) in the elderly is a serious injury. For patients with multiple existing comorbidities and frailty, the risk of complications, poor functional outcomes, and premature death may be higher. The treatment aims for immediate weight-bearing, preferably by hemiarthroplasty (HA). Previous research has focused on prosthetic joint infection and dislocation after HA. In this study, we aimed to describe the possible surgical consequences of periprosthetic fracture after HA.

Methods

HA patients with a periprosthetic fracture occurring between 2009 and 2019 were included in this retrospective cohort study. Patients were identified using local quality registers and systematic searches of the patient administrative system.

Results

We identified 56 periprosthetic fractures. A total of nine patients (16%) experienced complications related to the fracture. Of these, 42 patients were treated surgically for the periprosthetic fracture, and four patients required additional surgeries. Three of these four reoperated patients underwent two further operations, while one patient had a single reoperation. Within three months after surgery, three patients had died. Seventeen of the 56 patients (30%) required a permanently higher level of care after the periprosthetic fracture, and we observed an increased risk of new long-term care admissions (odds ratio (OR): 2.1; 95% confidence interval (CI): 1.1-4.2; p = 0.04). The 30-day mortality rate was 7%, and the one-year mortality rate was 27%. Most patients were treated surgically for their periprosthetic fractures. Regardless of treatment, 16% of patients experienced surgically related complications. Among the operated patients, 10% required additional surgical procedures. Nearly one-third of patients ultimately needed a permanently higher level of care following the injury.

Conclusions

In our cohort, we observed a low number of surgical complications, and only a few patients required reoperations. However, about one-third of the patients ultimately required a higher level of care.

## Introduction

Femoral neck fracture (FNF) in the elderly is a serious and common injury [1]. For the patient, it is a life-altering and serious event [2-4]. A low-energy FNF often indicates underlying frailty and comorbidities. Consequently, the risks of poor functional outcomes, surgical complications, and death are increased [5,6]. Most guidelines emphasize immediate weight-bearing as essential and recommend hemiarthroplasty (HA) as the preferred treatment method [7,8]. The three major orthopedic complications after HA are prosthetic joint infections, dislocations, and periprosthetic fractures.

Previous studies have reported an increased risk of inferior outcomes after HA when a prosthetic joint infection or dislocation occurs [9-12]. Recent studies have reported incidence rates for periprosthetic fractures after HA of 0.5-2% [13-15]. Several studies have evaluated the risk of periprosthetic fracture with respect to the fixation method. An RCT of 610 HA patients found periprosthetic fractures in 0.5% of the cemented group compared to 2.1% in the uncemented group [14]. A 2020 study from Kaiser Permanente Medical Group retrospectively compared over 12,000 HA patients with cemented versus uncemented femoral stems [16]. The study found a higher risk of aseptic revision among uncemented stems (3.0%) than cemented stems (1.3%), primarily due to periprosthetic fractures.

A few studies have described the consequences of experiencing a periprosthetic fracture after HA [13,17-19]. Findings in these studies vary from satisfactory recovery to substantial clinical consequences, including surgical complications and reoperations. Possible consequences of a periprosthetic fracture after HA include further surgical complications, reduced physical function, loss of independence, reduced quality of life (QoL), and death.

## Materials and methods

Patients

Patients were included from the local quality registers and systematic searches in the patient administrative system (Table 1). Patient charts and radiographs were reviewed to verify the diagnosis and extract data. Two hospitals contributed to the study: Oslo University Hospital and Bærum Hospital, each serving populations of 272,000 and 190,000 inhabitants, respectively, in southeastern Norway, and together treating 250-300 patients with femoral neck fractures per year.

**Table 1:** Search strategy and codes for identification in the patient medical records* ^*^[20,21] ICD-10: the International Classification of Diseases, 10th Revision; NCSP: the NOMESCO Classification of Surgical Procedures

Search priority	Diagnosis search (ICD-10)	Surgical procedure search (NCSP)	Description
1	T84.0		Complications of internal orthopedic prosthetic devices, implants, and grafts
2	M96.6		Fracture of bone following insertion of an orthopedic implant, joint prosthesis, or bone plate
3	T93.1		Sequelae of fracture of the femur
4		NFJ 4y	Internal fixation of the fracture of the femur using wire, rod, cerclage, or pin
5		NFJ 64	Internal fixation of the fracture of the diaphyseal femur using a plate and screws
6	S72	All NFCxx codes	“Fracture of femur” combined with all NFC procedure codes (secondary prosthesis in hip joint)
7	S72	All NFWxx codes	“Fracture of femur” combined with all NFW procedure codes (other reoperation in hip joint or femur)
8	M84.4		Pathological fracture, not elsewhere classified
9		All NFCxx codes	Secondary prosthesis in the hip joint

Aim of the study and study design

The study was designed as a retrospective cohort study and aimed to describe the complications and clinical consequences of periprosthetic fractures after HA. Reoperations, mortality, and changes in the level of dependency were recorded.

Data collection

A targeted search of the patient medical record system at each hospital was performed to identify eligible patients. The search was organized using International Classification of Diseases, 10th Revision (ICD-10) codes and NOMESCO Classification of Surgical Procedures (NCSP) codes related to fractures of the femur, osteosynthesis of the femur, and revision hip arthroplasty (Table 1) [20,21]. The search was designed to identify all patients who experienced a periprosthetic fracture after HA from 2009 to 2019. The search was conducted in April 2020, providing 16-132 months of follow-up after the periprosthetic fracture. Local quality registers and radiographs at the two institutions were also reviewed for supplementary information. HA patients with a periprosthetic fracture occurring either intraoperatively or postoperatively during the study period were included. Patients living outside the hospital catchment area were excluded, as they were unlikely to return for follow-up if a complication occurred.

We recorded age, sex, American Society of Anesthesiologists (ASA) score, and place of residence at admission [22]. We also registered surgical factors thought to influence the risk of a periprosthetic fracture: cemented or uncemented design, anatomical or straight stem, and surgical approach to the hip joint. Prodromal symptoms preceding the periprosthetic fracture and the trauma mechanism were recorded when noted in the chart. Fractures were classified according to the Vancouver fracture classification, and the chosen treatment was documented [23]. The applied treatment strategies were categorized as (1) non-operative treatment, (2) internal fixation alone, (3) stem revision, and (4) internal fixation with stem revision. We recorded 12-month outcomes for non-union, surgical site infection, dislocation, failure of internal fixation, subsequent fracture, hospital readmission for any cause, dependency (living independently at home, assisted living, or nursing home), changes in level of dependency, and mortality.

Statistical analyses

Statistical analyses were conducted using SPSS Statistics for Windows version 28 (IBM, Armonk, NY) and Stata SE17 (StataCorp, College Station, TX) with valid software licences for the University of Oslo. We performed an age-adjusted multivariate analysis (generalized estimating equations) on the change in dependency from the periprosthetic fracture to after the treatment. Significance level was set at 5%.

Ethical considerations

Both hospitals obtained the approval to carry out the study from their respective Hospital Data Protection Officer, and an exemption from patient consent was approved (reference number: 20/10506).

## Results

We identified 56 patients with a periprosthetic fracture (Table 2). Forty patients were female. Of note, 23 patients had received a cemented stem, and 33 patients had received an uncemented femoral stem. The median age at the time of the periprosthetic fracture was 85.5 years (Table 3). The median time from index surgery to periprosthetic fracture was 13 months (range: 0 to 263 months). Eighteen of the 56 patients had been in contact with a hospital between the index surgery and the periprosthetic fracture: three for dislocations, three for thigh or hip pain, and three for other orthopedic reasons, while nine patients had been admitted to a medical ward for stroke, urinary tract infections, pulmonary edema, or a deteriorated general condition. Forty-two of the 56 patients underwent surgical treatment for the periprosthetic fracture (Table 4). Four of the 42 surgically treated patients required further surgery.

**Table 2:** Baseline data at the time of femoral neck fracture* ^*^[22]. ^1^Data missing for three patients. ^2^Data missing for six patients. ^3^Data missing for one patient ASA: American Society of Anesthesiologists

Characteristics	Results
Female gender, n (%)	40/56 (71%)
Median age, years (range)	84 (55 to 96)
Median ASA score^1^	3
ASA group 1-2, n	22
ASA group 3-4, n	31
Residency^2^, n	
Home dwelling	33
Assisted living	8
Nursing home	9
Surgical approach^3^, n	
Direct lateral	20
Posterior	35
Femoral stem, implant name	
Uncemented	33
Corail	33
Cemented	23
Exeter	10
CPT	8
Charnley	3
Elite Plus	1
Bi-Metric	1

**Table 3:** Demographic data and results related to periprosthetic fracture treatment* ^*^[23]

Variables	Results
Median age at periprosthetic fracture, years (range)	85.5 (55 to 102)
Median time from index surgery to periprosthetic fracture, months (range)	13 (0 to 263)
Type of injury, n	
Fall from own height	48
Fall from a height higher than own height	2
Intraoperative fracture	5 (2 Vancouver B1 and 3 Vancouver A)
Pathological (atraumatic) fracture	1
Vancouver fracture classification and stem fixation, n	
A. cemented vs. uncemented	8 vs. 6
B1. cemented vs. uncemented	9 vs. 10
B2. cemented vs. uncemented	0 vs. 6
C. cemented vs. uncemented	6 vs. 11
Total cemented vs. uncemented	23 vs. 33
Readmittance, reoperations, and mortality	
Readmittance to hospital after treatment of periprosthetic fracture, n	26
Reoperation for non-union, n	1
Reoperation for prosthetic dislocation, n	2
Reoperation for ectopic bone formation, n	1
Median time to death after periprosthetic fracture, months (range)	15 (0 to 81)
30-day mortality, n (%)	4 (7)
1-year mortality, n (%)	15 (27)

**Table 4:** Vancouver classification and treatment* ^*^[23]

	N	Non-operative, n	Plate fixation, n	Stem revision, n	Stem revision and plate fixation, n	Suture anchor, n
Vancouver A	14	11	2	0	0	1
Vancouver B1	19	3	14	0	2	0
Vancouver B2	6	0	2	3	1	0
Vancouver B3	0	0	0	0	0	0
Vancouver C	17	0	16	0	1	0
Total	56	14	34	3	4	1

**Figure 1:**
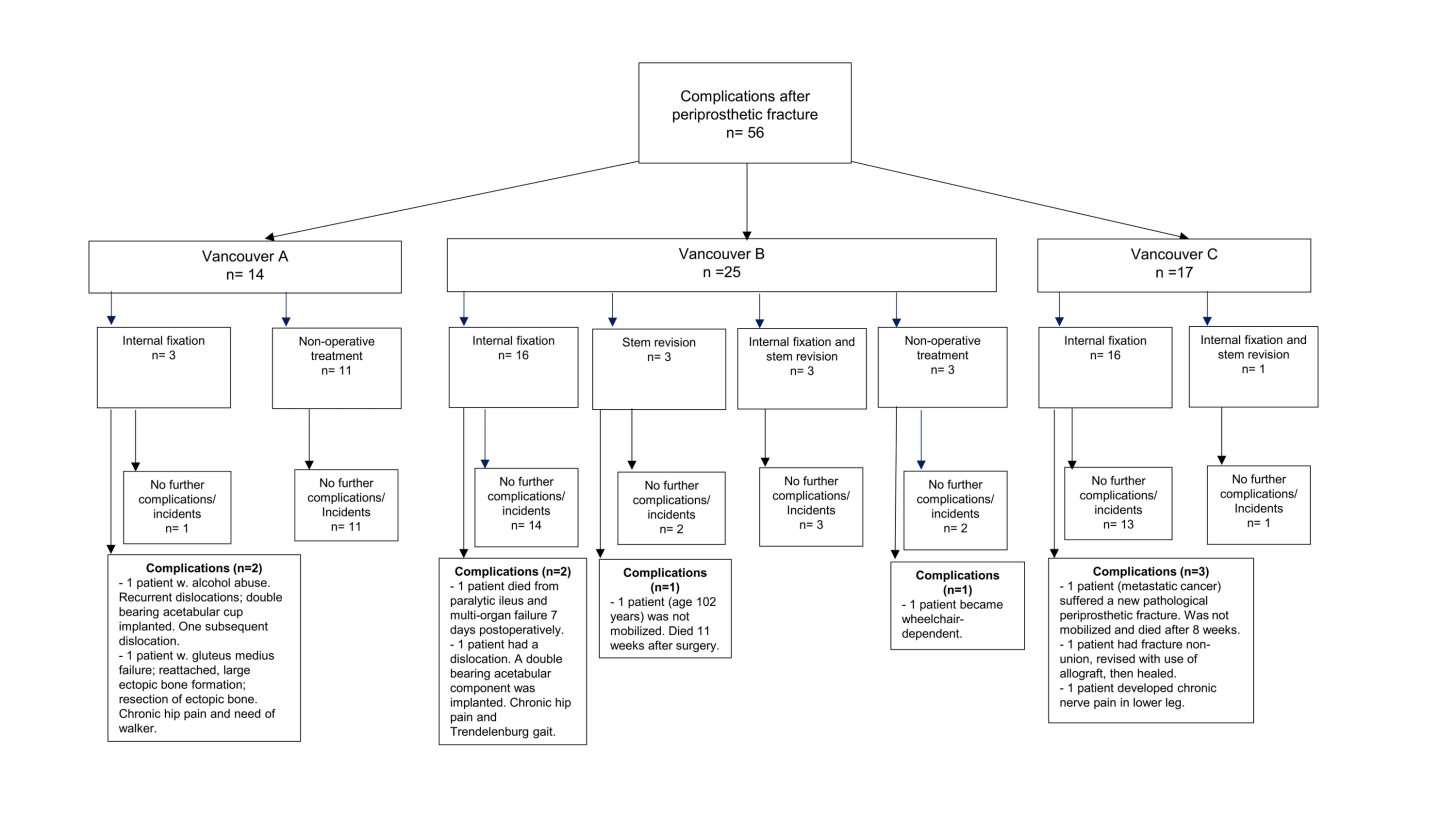
Complications after periprosthetic fracture in HA* ^*^[23] HA: hemiarthroplasty

We identified nine of 56 patients who experienced complications after the periprosthetic fracture (Figure 1). Twelve of 14 patients with a Vancouver A fracture experienced no further complications and had no confirmed poor outcomes. Twenty-one of 25 patients with a Vancouver B fracture (B1, B2, and B3) experienced no further complications. Fourteen of 17 patients with Vancouver C fractures had no complications.

We found an increase in dependency at the last follow-up (Table 5). Seventeen patients required a permanently higher level of care (long-term care or assisted living) after the periprosthetic fracture. After adjusting for age, we found an increased risk of new long-term care admissions (odds ratio (OR): 2.1; 95% confidence interval (CI): 1.1-4.2; p = 0.04).

**Table 5:** Change in dependency from periprosthetic fracture to the last follow-up Multivariate analysis adjusted for age, generalized estimating equations OR: odds ratio; CI: confidence interval

Status	At the time of periprosthetic fracture	Last follow-up		
			OR	P-value
Independent, n	23	13	Reference	-
Assisted living, n	10	6	2.5 (95% CI: 0.9-6.5)	0.067
Long-term care, n	22	34	2.1 (95% CI: 1.1-4.2)	0.04

## Discussion

Compared to the results after dislocations and infections in HA, we found few surgical complications following treatment of periprosthetic fractures. Only 10% of the patients who underwent surgery required further surgical procedures. Phillips et al. reported results comparable to ours [13]. Notably, 65 of 79 fractures were treated surgically. Of these, eight required further surgeries.

A study on surgical approaches in HA reported that 11 of 19 patients operated on for a dislocated HA needed more than one reoperation [10]. A study from the Swedish Arthroplasty Register found that two-thirds of the patients with a single HA dislocation had recurrent dislocations [24]. A study on prosthetic HA infections by Guren et al. found that reoperations were needed in 20 of 35 patients [9]. After dislocated and infected HA, 3/19 and 12/37 patients, respectively, underwent resection arthroplasties as the final procedure [9,10]. In our study, no patients underwent resection arthroplasty, similar to Phillips et al. [13], but one patient became wheelchair-bound after non-operative treatment. None of the 14 patients treated non-operatively for their periprosthetic fracture required subsequent surgery.

However, approximately one-third of the patients in our study needed a permanently higher level of care. Loss of independence reduces the quality of life for the patient and increases societal costs [25,26]. To our knowledge, no previous study has described increased dependency following a periprosthetic fracture after HA. Some of the patients would probably have needed more help regardless of their periprosthetic fracture, but it is not unreasonable to assume that the fracture contributed to the increased dependency.

Traditionally, Vancouver A fractures are managed non-operatively, while Vancouver C fractures are managed operatively with open reduction and fixation using a long locking plate. More variation is found in the treatment of Vancouver B-type fractures. It may be difficult to distinguish preoperatively between a fixed (Vancouver B1) and a loose stem (Vancouver B2/B3). Treatment protocols for periprosthetic femoral fractures are based on fractures after elective THA and advise stem revision in B2-type fractures without considering patient physiology, comorbidities, or the extent of the surgery [27]. In our study, two of the six patients with a Vancouver B2 fracture were treated with plate fixation only. One of these patients needed further surgery. Recent studies have advocated plate fixation alone for B2 fractures after HA in the elderly population [28,29]. We found a mortality rate after the periprosthetic fracture comparable to previous publications and to the mortality rates after primary HA for hip fractures [13,30].

Limitations and strengths

Our study has several limitations. The data were collected via a retrospective chart review and are vulnerable to inaccuracies and missing information. Even though we conducted an extensive search using possible diagnoses and procedure codes, we may have missed some patients. Regarding both ICD-10 and NCSP coding, we found several inconsistencies that made our search unreliable. Thus, we may have introduced selection bias into the cohort. For example, the use of NCSP codes to aid case identification may overestimate the proportion of patients who underwent surgical treatment. Some patients may have moved from our region or otherwise been treated for a periprosthetic fracture at other hospitals. Our study would have been methodologically stronger if we had included and followed all HAs performed during the same period to compare function and dependency between those who suffered a periprosthetic fracture and those who did not.

Nevertheless, the primary goal of the study was to determine how the patients were treated and how they managed postoperatively. Given the wide inclusion criteria and thorough chart review strategy, we believe we identified most, if not all, patients treated for a periprosthetic fracture at our two hospitals from 2009 to 2019. Thus, we believe we have a well-described study cohort and a nearly complete overview of the surgically related complications, as well as changes in dependency among the included patients.

## Conclusions

In our cohort, we found only a few surgical complications following the treatment of periprosthetic fractures after HA, and a few catastrophic outcomes. Compared to prosthetic joint infection and dislocation, a periprosthetic fracture may result in fewer surgical complications. However, about one-third of the patients in our cohort needed a permanently higher level of care.
